# Traditional complementary and alternative medicine (TCAM) for diabetic foot ulcer management: A systematic review

**DOI:** 10.1016/j.jaim.2023.100745

**Published:** 2023-07-11

**Authors:** Suman Kumar, Alakesh Bharali, Himangshu Sarma, Susankar Kushari, Sameeran Gam, Iswar Hazarika, Satyendra K. Prasad, Damiki Laloo

**Affiliations:** a Assam Science and Technology University, Guwahati, 781013, Assam, India; b Department of Pharmaceutical Chemistry, Girijananda Chowdhury Institute of Pharmaceutical Science, Girijananda Chowdhury University, Guwahati, 781017, Assam, India; c Department of Pharmaceutics, Girijananda Chowdhury Institute of Pharmaceutical Science, Girijananda Chowdhury University, Guwahati, 781017, Assam, India; d Department of Pharmacognosy, NETES Institute of Pharmaceutical Science, Mirza, 781101, Assam, India; e Department of Pharmacology, Girijananda Chowdhury Institute of Pharmaceutical Science, Girijananda Chowdhury University, Guwahati, 781017, Assam, India; f Department of Pharmacognosy, R.T.M. Nagpur University, Nagpur, 440033, India; g Phytochemical Research Laboratory, Department of Pharmacognosy, Girijananda Chowdhury Institute of Pharmaceutical Science, Girijananda Chowdhury University, Guwahati, 781017, Assam, India

**Keywords:** Diabetic foot ulcer, Traditional complementary and alternative medicine, Medicinal plant extracts, Polyherbal formulation, Clinical aspect, Molecular mechanism

## Abstract

Diabetic Foot Ulcers (DFUs) are a devastating micro-vascular complication of diabetes with an increased prevalence and incidence and high rate of morbidity and mortality. Since antibiotics are frequently used to treat DFU, managing the condition has proven to be extremely challenging and may eventually lead to the development of antibiotic resistance. Scientists from around the world are working to develop an alternative solution to the problem of drug resistance by exploring complementary and alternative medicines that may be obtained from natural sources. Hence, the review aims to comprehensively report the information on the natural treatments and therapy used to manage DFU. All of the information described in the current study was gathered from electronic scientific resources, including Google Scholar, PubMed, Scopus, Science Direct, and Springer Link. Findings from the current review revealed the pre-clinical and clinical utility of 18 medicinal plants, 1 isolated compound, 7 polyherbal formulations including herbal creams, a few micronutrients including vitamins and minerals, insect products such as propolis, honey and, Maggot debridement therapy for the treatment and management of DFU. Natural therapies possess better efficacy, low cost, and shorter duration of treatment when compared with the conventional treatments; hence, all information made available about them is crucial to alter the direction of treatment. Furthermore, the data presented in this review are up to date on the potential efficacy of natural complementary medicines for alleviating DFU problems in *in vitro* and *in vivo* tests, as well as clinical studies.

## Highlights

• The review describe the utilization of natural therapy for the treatment of DFU.
• 18 medicinal plants, 7 herbal formulations and few insect products were reported to be used in DFU.
• The review also discusses the clinical aspects of ethnomedicinal plants for DFU management.

## Graphical abstract

**Figure:**
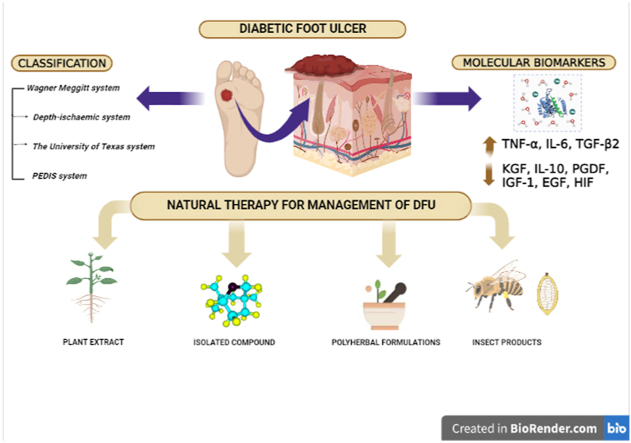

## 1 Introduction

Diabetes mellitus (DM), a chronic endocrine condition, is distinguished by persistent hyperglycemia caused by the body's inability to make or use insulin efficiently. It is one of the world's most devastating diseases, and it is frequently coupled with various secondary consequences [1]. Diabetic foot ulcers are such secondary problems connected with diabetes, and in extreme situations, the lesions can lead to catastrophic physical repercussions such as peripheral neuropathy, as well as motor neuropathies, which produce foot deformity, local pressure, ulceration and ultimately limb amputation [2,3]. It also has an impact on the patient's social and economic position, including facial embarrassment, social isolation, and unemployment. Several studies have found that more than a quarter of the diabetic population will suffer from DFU complications in their lifetime [3,4]. To minimize ulcer recurrences, patients were urged to take good care by wearing suitable therapeutic footwear and maintaining routine foot care [5]. To avoid amputations and improve the patient's quality of life, it is critical to implement a stringent ulcer prevention and treatment program, as well as thorough infection control [3]. Furthermore, the treatment of illnesses is difficult and costs billions of dollars in direct medical expenses due to an increased frequency of hospital stays and drug intervention [6]. The International Working Group on the Diabetic Foot (IWGDF) recognized *Pseudomonas aeruginosa, Escherichia coli, Staphylococcus aureus*, and *Streptococcus pyogens* as the main microorganism responsible for the development of DFU [3]. Hence, the drugs of choice for the management of DFUs are oral antibiotics regimes including dicloxacillin, cephalexin, and clindamycin. However, prolonged use of such medications might result in the microorganisms developing antibiotic resistance, a critical issue that exists in the present day. The widely accepted gold standard therapy for the management of DFU is exfoliation of the lesion, off-loading the ulcer, control the infected area, and revascularization procedures when needed. A total contact cast (TCC) is currently another commonly used gold standard for the therapy of DFU and is applied to the patient's foot to relieve pressure on the ulcer region [7]. Other methods, including negative pressure wound therapy (NPWT), hyperbaric oxygen therapy, and the use of advanced wound care products, have also been suggested as helpful supplemental therapies [8]. Because of the limited treatment availability, drug resistance, high cost and, other serious consequences, diabetic foot syndrome is becoming an alarming issue. To overcome such problems, scientists from all over the globe attempt to find an alternative way to fill the gaps of drug resistance by researching complementary medicines available from natural sources. Hence, the current review aims to report the detail available information on the plant, animal and, insect products and some minerals for the management of DFU.

## 2 Methodology

**Fig. 1:**
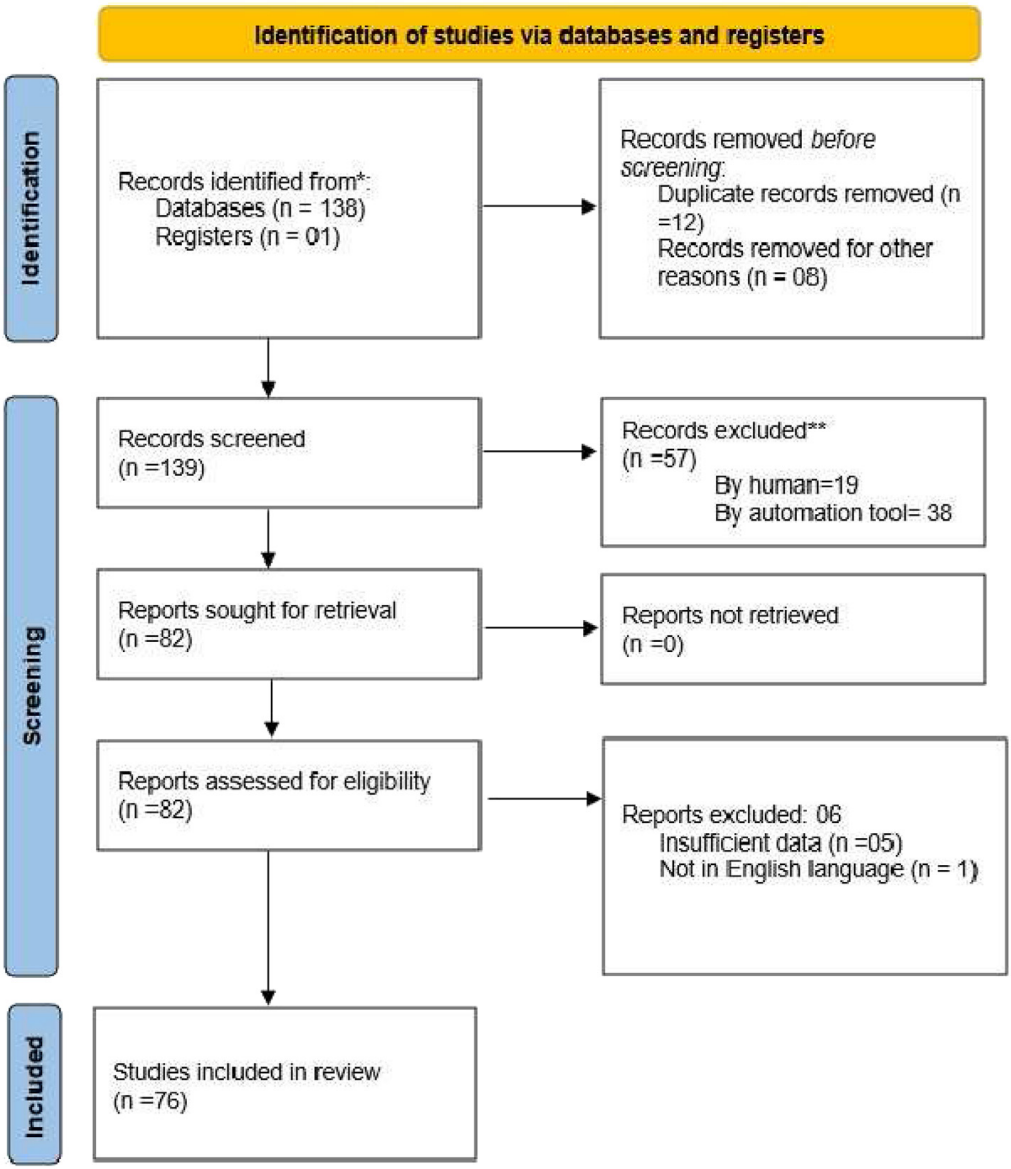
PRISMA flow diagram of the studies selected for analysis.

All the applicable information regarding diabetic foot ulcer were collected from published literature in the English language. The electronic databases used for the collection of relevant information include PubMed, Scopus, Google Scholar, Science Direct, and Springer Link. Among the search terms used were “Diabetic foot ulcer” AND “Herbal treatment; Traditional medicine; Medicinal plants; Active phytoconstituents”; “Diabetic foot infections” AND “Herbal treatment; Traditional medicine; Medicinal plants; Active phytoconstituents”; “Diabetic foot ulcer” AND “Pathogenesis” “Diabetic foot ulcer” AND “Classification system”; "Diabetic foot ulcer/Diabetic foot infections” AND “Insect/animal products", with no word limits. Inclusion criteria include the availability of English-language data, herbal and natural remedies for diabetic foot ulcers, the prevalence of the diseases, classifications, and risk factors associated with them. Exclusion criteria includes (a) Articles published in foreign languages other than English, (b) Articles with ambiguous or inadequate data, (c) Pharmaceuticals derived from semi-synthetic and synthetic chemicals, and (d) Articles that involves routine diabetic wounds rather than diabetic foot ulcers. The major perspective of the study is to accumulate all the natural therapy including herbal, polyherbal, and animal products used for the treatment of DFU. In addition, books and abstracts which fulfilled the inclusion criteria were included in the review paper. The PRISMA flow diagram for the source selection process is shown in Fig. 1.

## 3 Prevalence and incidence of DFU

Increasing cases of diabetes mellitus, especially type-2 diabetes, have raised public health concerns on a global scale. Globally, there were 425 million persons with DM in 2017, and by 2045, the number is expected to rise to 629 million [9]. It is considered a major economic burden for all countries, but developing countries suffer the worst of it because they account for more than 80% of all cases [6]. Incidence of DFU is mostly observed in patients having an extended history of diabetes and the prevalence rate is between 5.3% and 10.5% [10]. The International Diabetes Federation in 2015, reported that males are more prone to the diseases in comparison with female populations. Furthermore, approximately 9.1 million to 26 million diabetic patients experience foot ulcers every year globally [11,3]. One large prospective study reported by the IWGDF in 2019 revealed that 46% of ulcers had healed after one year, 10% had recurred, 15% had died, and 17% required amputation of the lower extremity. According to a survey reported in 2017, the global average cost of healthcare for patients, including primary care, outpatient treatment, and special inpatient hospital care, is US $16920. In the United States, the overall medical expenses for treating diabetic foot disease, in addition to the expense of treating DM alone, range from $9 to $13 billion. In middle-income countries particularly India and China, the cost of management of DFU is $19599 per patient and $ 21,372 per patient [12,13]. Additionally, a meta-analysis has suggested that the global prevalence of DFU was approximately 6.3%. The study also stated that the highest prevalence was observed in North America (13.0%), while Australia reported the lowest prevalence of 3.0%. Moreover, the prevalence was relatively higher in Africa (7.2%) as compared to Asia (5.5%) and Europe (5.1%) [14].

## 4 Classification system for assessing DFU

IWGDF have developed a systematic categorization system to aid research in DFU. This approach classifies DFUs based on their size, depth, infection, feeling, and degree of perfusion [15]. Understanding appropriate categorization is critical for assisting in the development of DFU treatment choices. To assess and determine diabetic foot severity, there are multiple classification systems available today, including the Wagner System, the University of Texas System, the Depth Ischemic Classification System, and the PEDIS System, which attempt to cover multiple ulcer parameters such as size, depth, and depth of the foot, among others, etc.

### 4.1 Wagner–Meggitt system

The Wagner-Meggitt method is the most widely used DFU categorization approach, which categorises DFUs based on the degree of ulceration and gangrene severity. This method divides foot lesions into five grades, ranging from 0 to 5. A high-risk foot with no active lesions is designated as grade 0, whereas gangrene over the foot is designated as grade 5. Only grade three is concerned about infection. The disadvantage of this approach is that it does not address ischemia or neuropathy [8].

### 4.2 Depth-ischaemic classification system

This system is based on the Wagner-Meggitt categorization system. The goal of this technique is to provide exact and scientific classification of DFU, to make it simpler to separate wounds from foot vascularity, to clarify distinctions between grade 2 and grade 3, and to ease treatment–grade correlation [16].

### 4.3 The University of Texas System

Ischemia and lesion depth are factors considered by the University of Texas San Antonio classification system. In reality, it is an upgraded version of the Wagner system. In this approach, each Wagner system grade is divided into phases based on the infection, ischemia, or combination of factors. This classification, however, is the most confounding and difficult due to the various grades and phases. It can also be challenging to use on a daily basis since it must be used in conjunction with other clinical data rather than as a stand-alone tool for selecting treatment options or comparing research findings [17].

### 4.4 PEDIS classification

The PEDIS classification was developed by the IWGDF to alert persons at risk of foot illness about the hazards of peripheral vascular disease and neuropathy. Perfusion, extent, infection, depth, and sensation are the five features that make up the DFU PEDIS categories. It has a four-point scale for diagnosing infections and appears to predict DFU outcomes depending on risk variables [16,18].

## 5 Pathogenesis of DFU

**Fig. 2:**
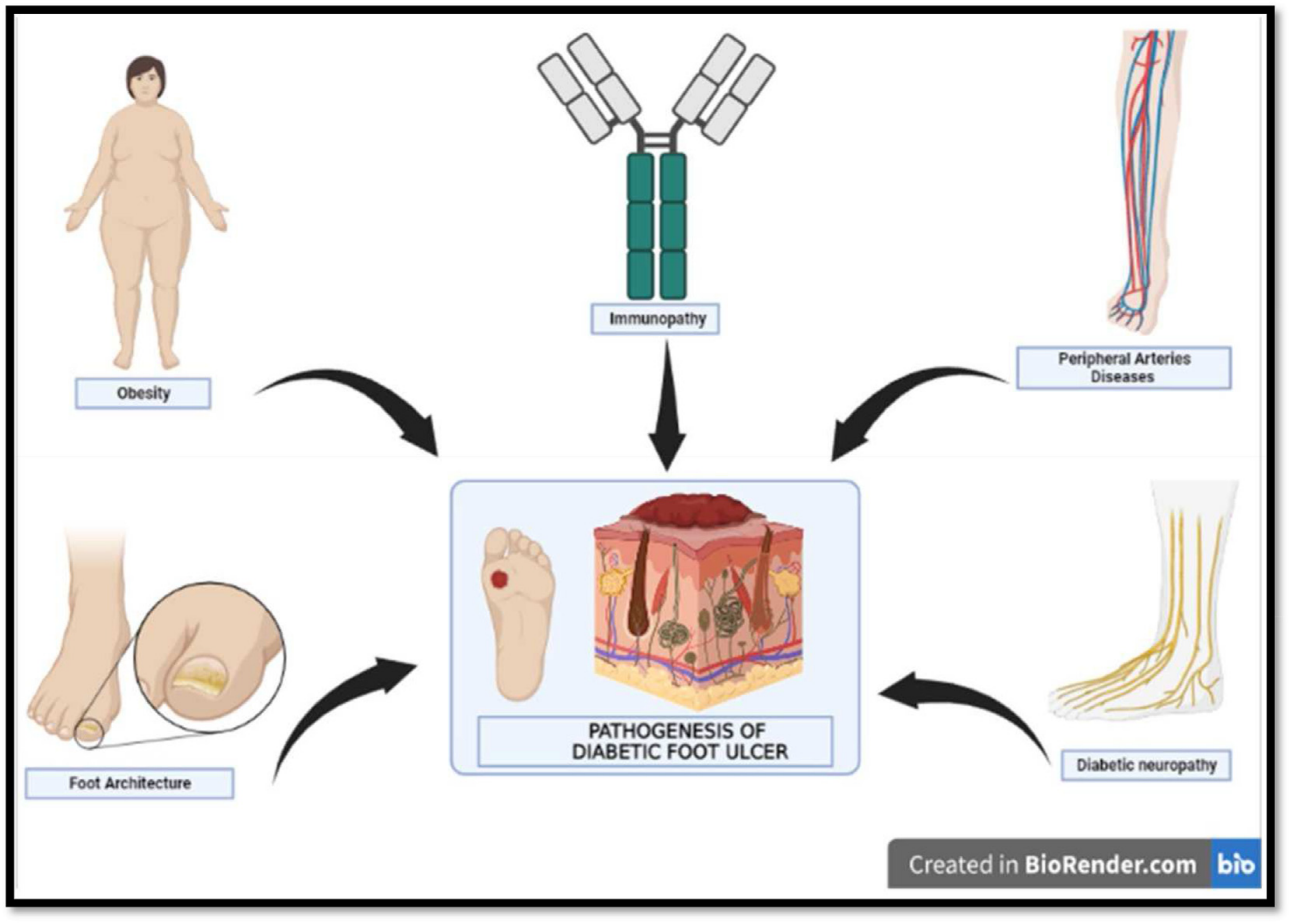
Pathogenesis of diabetic foot ulcer.

Several risk factors, such as peripheral vascular disease, diabetic neuropathy, biomechanical abnormalities, past foot ulcers, poor glucose management, long-term diabetes, etc., are responsible for the development of DFU as a result of mild trauma or foot deformities (Fig. 2). Diabetic neuropathy, insufficient circulation, and infection susceptibility are three of the key triggers contributing to DFU [10,19].

### 5.1 Diabetic neuropathy

More than 60% of diabetic neuropathy and 50–75% of non-traumatic amputations are caused by DFUs [19,20]. In this clinical situation, the creation of a few enzymes, such as aldose reductase and sorbitol dehydrogenase, is decreased, limiting the activity of glucose-6-phosphate dehydrogenase and hence constraining nicotinamide adenine dinucleotide phosphate (NADPH) synthesis from nicotinamide adenine dinucleotide (NAD). As a result, sorbitol and fructose gets converted from glucose, which reduces myoinositol production in nerve cells even further. The foot may have diminished peripheral sensibility and epidermal cracks as a result of nerve cell damage [21,3].

### 5.2 PAD: peripheral arteries diseases (PAD)

PAD is caused by hyperglycemia, which causes endothelial cell dysfunction due to reduced vasodilator production [3,22]. It is a disorder characterised by atherosclerotic stenosis of the lower limb arteries [22]. It primarily raises plasma thromboxane A2, which causes hypercoagulation of plasma and increases the risk of ischemia and ulceration. Endothelial cell proliferation is affected by changes in basement membrane thickness, an increase in blood viscosity, smooth muscle cell proliferation, changes in microvascular tone, decreased antioxidant activity, and decreased angiogenesis [3,20]. Noninvasive vascular screening tests, such as Ankle-Brachial Index (ABI), are most useful in detecting PAD [23].

### 5.3 Immunopathy

Immunopathy in diabetics is characterised by a possible slowing of the inflammatory response as well as diminished susceptibility to infections. Diabetic people have a reduced immune response as compared to non-diabetics. Reduced neutrophil functions, chemotaxis, phagocytosis, and a reduced T-cell response have all been found in patients. It also affects leukocyte capacity, macrophage morphology, and the production of pro-inflammatory cytokines, all of which contribute to the severity of DFU [23].

### 5.4 Obesity

Obesity is one of the most commonly reported uncertain risk factors for DFU development [14]. Vela and his colleagues investigated the impact of weight gain on peak foot pressure, which may contribute to the development of DFU, by measuring mean peak plantar foot pressures in both sexes. The study concluded that weight gain raises pressures in the first metatarsal, lesser metatarsal, midfoot, and heel areas. The study also revealed that adding 9.1 and 18.2 kg of body weight increased average peak foot pressure by 5% and 19% in males and 9% and 25% in women, respectively, when confounding variables were controlled [24,14].

### 5.5 Foot architecture

The unique architecture of the foot is mainly accountable for its target in DFU. The most usually infected soft tissues are the plantar tendons, muscle sheaths, aponeurosis, and fascia. A sterile metal probe can be placed into the ulcer region to detect the existence of an infected bone. Furthermore, CT scans, MRI, radioisotope scans, and plain radiographs give high-resolution information on bone and soft tissue infection [23].

## 6 Molecular mechanism associated with DFU

**Fig. 3:**
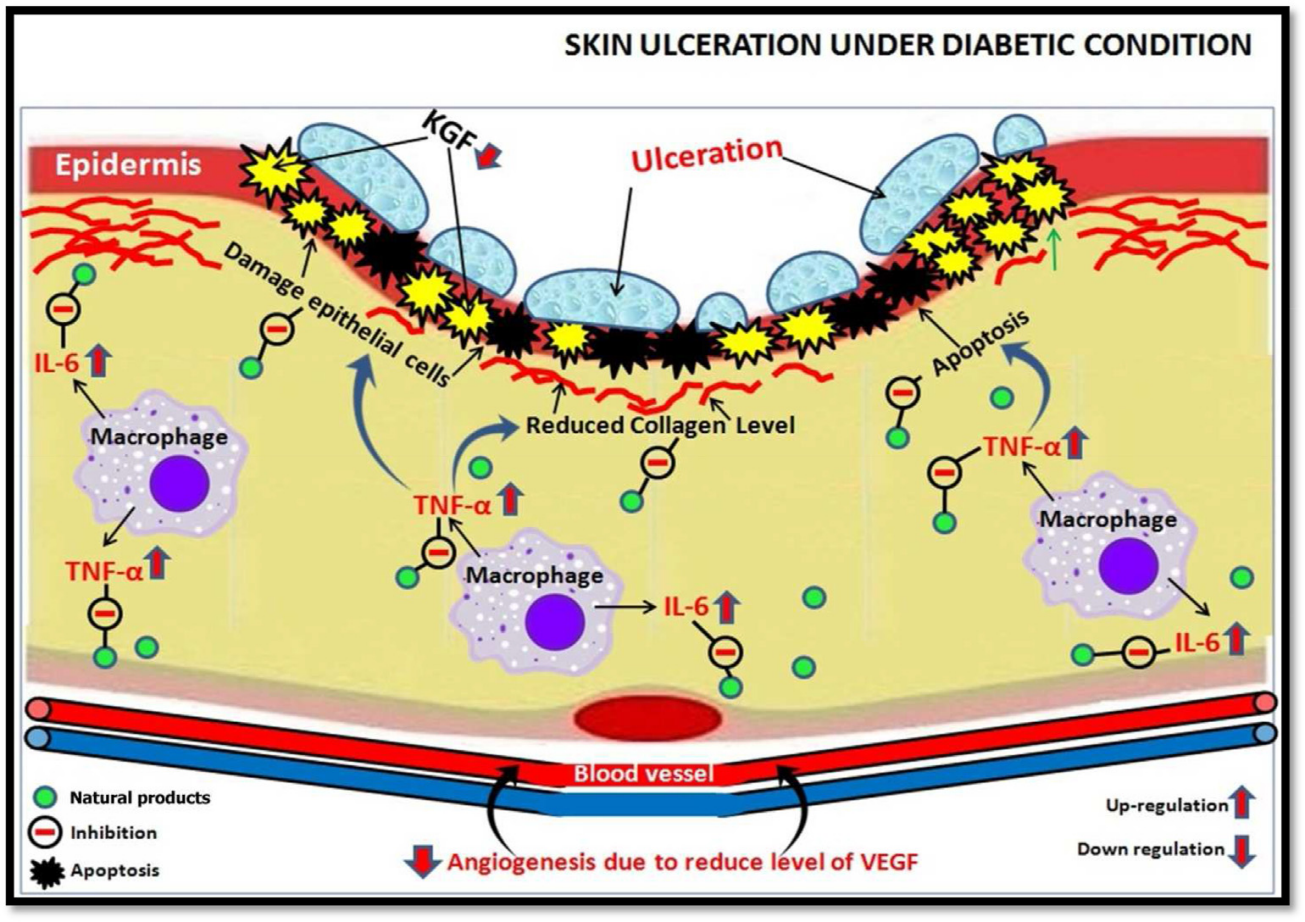
Role of natural products in regulating the levels of molecular biomarkers associated with DFU.

The prolonged wound healing process of diabetic patients is largely due to several physiological factors, including decreases in collagen deposition linked to lower levels of endogenous growth factors like leukocyte chemotactic and angiogenic factors [25,26]. DFU impairs wound healing by preventing clot formation, angiogenesis, reepithelialization, nerve cells regeneration, and the production of extracellular matrix (ECM). In this diseased state, several molecular components, including receptors, proteolytic enzymes, and neuropeptides, have been altered, resulting in protracted wound healing [27]. When damaged blood arteries on wounds are sealed, the hypoxia-inducible factor (HIF) complex is activated, which is directly implicated in the production of molecular factors such as vascular endothelial growth factor (VEGF). Transforming growth factor-3 (TGF-3)upregulation in the epidermis decreases TGF-1 expression, which is important for suppressing macrophage activity. Furthermore, anti-inflammatory cytokinins such as interleukin (IL)-10 and keratinocyte growth factor (KGF) are downregulated, whereas IL-6 and tumour necrosis factor (TNF-α) are elevated (Fig. 3). There is also a decline in other angiogenesis-promoting growth factors, such as insulin-like growth factor-1 (IGF-1), platelet-derived growth factor (PDGF), epidermal growth factor (EGF), IL-8, and nerve growth factor (NGF), in DFU, which results in inadequate blood flow. Due to the downregulation of growth factors like IGF-1, TGF-1, and PDGF, as well as collagen and glycosaminoglycan (GAG), poor extracellular matrix (ECM) is always related to DFU [27]. Growth factors, anti-diabetic medications, urokinase, botanicals, statins, dalteparin, and bio-agents such acid peptide matrix are only a few of the molecular variables that have been linked to the complicated pathophysiology and slow healing of DFU [3,28].

## 7 Natural approach for the treatment and management of DFU

Despite the disease's great incidence, it is still unknown how to manage and treat DFU. Very little information was accessible to help regulate and manage the medical state using natural therapies. There have been reports of a few plants (Table 1), isolated components, insect products, and polyherbal formulations having substantial effectiveness against DFU which is represented as follows.

**Table 1:** Summary on the list of medicinal plants and their pharmacological effects in the management of DFU.

SL. No	Scientific Name	Family	Common Name	Type of Studies	Plant parts and extracts	Pharmacological Action	Reference
1.	*Abutilon indicum* L.	Malvaceae	Indian mallow	*In vitro*	Fruit ethanolic extract	Extract loaded in solid lipid nanoparticle exhibit significant antimicrobial activity against various gram positive and gram-negative bacteria isolated from DFU.	[45]
2.	*Actinidia deliciosa* (A.Chev.) C·F.LiangandA.R.Ferguson	Actinidiaceae	Kiwifruit	Clinical trial	Fruit pure extract	The extract significantly reduced the surface area of the foot ulcer as compared to the control group. It increases the amount of collagen and granulation tissue in kiwifruit-treated individuals.	[46]
3.	*Ageratinapichinchensis* (Kunth) R.M.KingandH.Rob.	Compositae	–	Clinical trial	Aerial part, 7:3 hexane-ethyl acetate extract	The plant extract significantly healed diabetic foot ulcer completely after 24 weeks of treatment.	[47,48]
4.	*Aloe vera* Mill.	Liliaceae	Aloe	*In vivo*	Aerial part ethanolic extract	Parenchyma extract of *Aloe*showed remarkable ulcer healing activity on experimental animals.	[49]
				Case report	Aerial part ethanolic extract	Topical application of the plant after continuous IR radiation therapy significantly decreases the ulcer healing time.	[50]
				Clinical trial	Aerial part ethanolic extract	*Aloe vera* dressing significantly reduces the ulcer area.	[51]
7.	*Aristolochia indica* L*.*	Aristolochiaceae	Indian Birthwort, Snakeroot	*In vitro*	ZnO nanoparticles of Aqueous extract	ZnO nanoparticle of aqueous extract of the plant possesses significant antimicrobial activity which aids in bacterial cell death.	[52]
8.	*Azadirachta indica* A.Juss.	Meliaceae	Neem	Clinical trial	Leaf aqueous extract	Extract of the plant significantly increases the diabetic ulcer healing activity on topical application.	[53]
9.	*Coffea canephora*	Rubiaceae	Coffee	Clinical trial	Fruit aqueous extract	Coffee powder extract has significantly increased the speed of granulation tissue and also the ability to fight infection in the wounds after 3–4 days continuous treatment.	[54]
10.	*Davilla nitida* (Vahl) Kubitzki	Dilleniaceae	Chaparro	*In vitro*	Bark ethanolic extract	Extract of the plant showed a remarkable antimicrobial activity against multidrug resistant bacteria isolated from diabetic foot ulcer.	[55]
11.	*Lavandula angustifolia* Mill.	Lamiaceae	Lavender	*In vitro*	Leaf, 70% Ethanol extract	The plant extract was found to have considerable MIC value against DFU pathogenic bacteria such as against methicillin-sensitive and methicillin-resistant strains of *Staphylococcus aureus* in comparison with standard antibiotic gentamycin.	[56]
12.	*Melilotus officinalis* (L.) Pall.	Fabaceae	Yellow Sweet Clover	Clinical trial	Flower extract	The plant significantly reduces the diabetic foot ulcer area after continuous treatment.	[39]
13.	*Myrtus communis* L.	Myrtaceae	Myrtle	Case report	Fruit aqueous extract	The fruit part of the plant potentially heals diabetic foot ulcers by inhibiting inflammatory response, and oxidative stress.	[57]
14.	*Onosmamicrocarpum* DC.	Boraginaceae	Onosma	*In vivo*	Root hexane extract	Root part of the plant showed complete healing of diabetic foot ulcer after 20 days continuous treatment.	[58]
15.	*Quercus infectoria* G. Olivier	Fagaceae	Galls, Mecca Galls, Magic Nuts	Clinical trial	Fruit ethanol extract	The plant significantly reduces the wound size after continuous treatment of 8 months.	[59]
16.	*Rehmanniaglutinosa* Libosch	Scrophulariaceae	Chinese foxglove	*In-vivo*	Whole plant aqueous extract	Extract showed remarkable reduction of ulcer area by increasing the release of vascular endothelial growth factor (VEGF).	[60]
17.	*Strychnosnux-vomica* L.	Loganiaceae	Nux-vomica	*In vitro*	ZnO nanoparticle of Aqueous extract	ZnO nanoparticle of extract showed remarkable antimicrobial activity against multidrug resistant bacteria isolated from DFU.	[61]
18.	*Teucrium polium*	Lamiaceae	Felty germander	*In vivo*	Fruit ethanolic extract	10% ointment of the extract showed highest ulcer healing activity.	[62]
19.	*Tinospora cordifolia* (Willd.) Miers	Menispermaceae	Gurjo	Clinical trials	Fruit aqueous Extract	The clinical study signified that adjuvant therapy of *T. cordifolia* improves the healing of DFU by reducing debridement.	[63]
20.	*Urtica dioica* L.	Urticaceae	Nettle	*In vitro*	Leaf, 70% ethanol extract	The plant extract was found to have considerable MIC value against DFU pathogenic bacteria such as against methicillin-sensitive and methicillin-resistant strains of *Staphylococcus aureus* in comparison with standard antibiotic gentamycin.	[56]

### 7.1 Medicinal plants

Numerous plant and herb species with wound-healing properties under diabetic state have been discovered as a result of ethnobotanical research in Africa and other developing nations. Based on the evidence many researchers have scientifically validated the use by using various experimental models. The article lists a total of 18 medicinal plant extracts that can treat DFUs; of these, six plants were reported to be tested in *in vitro* assay, four plants were tested in *in vivo* models, two plants were reported in case studies, and eight plants were involved in clinical tests. *Ageratina pichinchensis* (Kunth), *Aloe vera* Mill., *Actinidia deliciosa* (A.Chev.), *Aristolochia indica* A. L. *Azadirachta indica* A. Juss., *Coffea canephora* and *Melilotus officinalis* (L.) Pall, are a few medicinal plants that possess a remarkable DFU healing activity. A detailed explanation of the pharmacological activity, plant parts, and method of application is listed in Table 1.

### 7.2 Isolated compound (naringin)

Chemically, naringin, also known as 4′,5,7-trihydroxy flavanone 7-rhamnoglucoside, is a flavanone glycoside derived from various grape and citrus fruits, which has many therapeutic applications. Naringin have a wide spectrum of pharmacological activities including anti-inflammatory, anti-ulcer, anticancer, antioxidant, antiatherogenic, neuroprotective, and hepatoprotective properties [29,30]. Kandhare and associates in 2016, performed an *in vivo* experiment on DFU by using streptozotocin (STZ, 55 mg/kg, i. p.) induced diabetic rats. The healing potential of naringin (20, 40, and 80 mg/kg, p. o.) was investigated in rats with surgically implanted wounds on the dorsal surface of their hind paws against standard insulin (10 IU/kg, s. c.) and tetrachlorodecaoxide (TCDO) (1 drop, twice a day, topically) for 16 days. After analyzing all of the statistical data, it was determined that -naringin treatment at 40 and 80 mg/kg doses results in significant (P < 0.05) DFU healing. In addition, naringin leads to the upregulation of mRNA expression of growth factors such as VEGF-c, TGF-β, and IGF-1, as well as the downregulation of mediators of inflammation including TNF-α, IL-1β, and IL-6 [31].

### 7.3 Polyherbal formulations

In India, the concept of polyherbal formulation has been highlighted in Ayurvedic literature “Sarangdhar Samhita” since 1300 A. D. It is mainly based on combining several medicinal herbs to achieve a better therapeutic effect. Similarly, it requires a low dose to achieve desirable pharmacological activity which ultimately reduces the risk of deleterious side effects in humans [32]. Many other traditional practices, like traditional Chinese medicine, traditional European medicine, traditional Korean medicine, traditional African medicine, etc., also use the concept of polyherbal formulations for preparing herbal products [33].

#### 7.3.1 Jing Wan Hong ointment

The ointment comprises thirty different Chinese herbs that are intended to stimulate blood circulation in order to disperse blood stasis, clear heat and moisture, and decrease swelling through detoxification. Out of the 30 varieties of Chinese herbs used, the most common ingredients present in the ointment include *Angelica dahurica*, *Boreol, Chinese lobelia, Pangoli, Radix ampelopsis*, *Rhizoma atractylodis*, *Red Peony* root and *Rhizoma* *ligustici Chuanxiong*. Using Wistar rats induced with streptozotocin to develop diabetes and sciatic nerve damage to induce DFU, the study planned to evaluate the efficacy and mechanisms of healing of the ointment. Jing Wan Hong ointment appeared to reduce the severity of foot ulcers in diabetic rats that had suffered initial nerve damage. The size of the foot ulcer and the Wagner grade were both lowered after seven days of therapy. Diabetic rats with foot ulcers healed almost completely after 21 days of therapy. Additionally, this effect appeared to be mediated by an increase in PDGF mRNA expression, but there was no impact on TGF, VEGF, or FLT-1 (Fms Related Receptor Tyrosine Kinase 1) mRNA expression [34].

#### 7.3.2 Ulcer oil (UO)

Ulcer oil is a Chinese herbal medicine that is used externally to treat wounds and purge toxins. It contains chiefly *Angelica* *dahurica* along with *Cortex* *phellodendri*. The pharmacological activity of these plants justifies the use of such Chinese herbal formulations in DFU. Jia and co-workers (2018), investigated the use of ulcer oil in DFU by using streptozotocin-induced diabetic rats. Additionally, they have explored the mechanism by which it is accelerating the healing of ulcers with the help of the western blotting technique. After completion of the study, it was found that ulcer oil is capable of inhibiting Protein-tyrosine Phosphatase 1B (PTP1B) and advanced glycosylated end products (AGEs) expression and enhancing VEGF and PDGF expression contributing to ulcer healing [35].

#### 7.3.3 Holoil gel

Holoil gel is widely used in Italy and is made up of a mixture of *Hypericum perforatum* flower extract and *Azadirachta indica* neem oil. In 2013, Iabichella and associates performed a case study on the 72 years old DFU patients with type 2 diabetes. During the experiment, the lesions were cleaned and Holoil gel was applied two times a week for 4 months. After continuous observation, it was found that the ulcer showed granulation tissue and wound-healing epithelization. Moreover, it significantly reduces the size of the ulcer area which implies the use of this herbal gel for the treatment of DFU [36].

### 7.4 Herbal creams

#### 7.4.1 Bensal HP

*Bensal HP* is a topical ointment used to treat ulcers and wounds with recalcitrance. Active ingredients are salicylic acid (3%) and benzoic acid (6%) in a polyethylene glycol base and *Quercus rubra* bark extract (3%). This formulation was developed by Harry Stanley, and permission was obtained from Food and Drug Administration (FDA) as an over-the-counter (OTC) drug for treating certain eczematoid skin conditions including inflammation and irritation. After observing the therapeutic potential against a wide range of inflammatory diseases, Jacobs and Tomczak have decided to perform clinical trials for the formulation against DFU and compared with positive control taken as silver sulfadiazine cream (SSC) for the treatment of Wagner grade 1 or 2-foot ulcers. The human trial showed significant results in comparison with standard SSC for the treatment of DFU (Wagner grade 1 or 2). The study included 40 diabetic patients with ulcers with a diameter of 3 cm, regardless of type of diabetes. There was no significant difference between the Bensal HP group and the SSC group at the start of the investigation, but at the end of the study, the wound diameter of the Bensal HP treated group had shrunk to 72.5%, compared to 54.7% in the SSC group, which is statistically significant (P = 0.059) [37].

#### 7.4.2 Semelil (Angipars™)

Semelil (Angipars™) is a natural herb extract of the *Melilotus* *officinalis* that has been proposed for the treatment of DFU [38]. This activity is primarily driven by coumarins and flavonoids. In 2008, Larijani and associates conducted a multicenter clinical study to evaluate the effectiveness of Semelil (Angipars™) to accelerate the DFU healing. All 25 patients were divided into two groups, comprising a treatment group that received Semelil along with conventional therapy and another group (control group) that received only conventional therapy. After 4 weeks of treatment, the percentage of decreases in surface area fluctuates significantly in the treatment group with 64% in comparison to the control 25%. Similarly, the mean ulcer area in the treatment and control groups were 479.93 ± 379.75 mm^2^ and 766.22 ± 960.50 mm^2^, which reduces to 198.93 ± 143.75 and 689.11 ± 846.74 after treatment, respectively [39]. Additionally, in another study, the dose-limiting toxicity (DLT) and the maximum tolerated dose (MTD), in a Phase I clinical trial for DFU has been performed for Semelil (Angipars™) [40]. In the study they have observed that foot ulcer dramatically improves up to the dose of 10 cc per day and phlebitis at the infusion site was observed with a dose of 13.5 cc per day. The study revealed that the maximum tolerated dose (MTD) for Semelil (Angipars™) was achieved at 10 cc/day and dose-limiting toxicity (DLT) showed phlebitis in the injection vein. Semelil (Angipars™) does not appear to have significant side effects or toxicity, suggesting that it could become a routine part of DFU therapy.

#### 7.4.3 Other herbal cream

Kuo et al. 2012 utilized a single-centre, controlled, open-label study involving twenty-four patients suffering from Wagner grade 3 foot ulcers to assess the healing effects of an herbal cream containing *Plectranthus amboinicus* (Lour.) Spreng (Lamiaceae) and *Centella asiatica* (L.). Urban (Umbelliferae). All patients were divided into two groups, one for the herb cream and the other for hydrocolloid fiber dressings. There was no statistically significant difference in wound size between the herbal cream and hydrocolloid dressing groups after 7 and 14 days of monitoring. In addition, herbal cream showed a slight improvement among patients compared to hydrocolloid fiber without any statistical significance. It was found that herbal cream could be used instead of hydrocolloid fiber dressing in treating DFU [2]. In another study, Mahboubi and associates studied the effectiveness of a herbal cream containing *Pelargonium graveolens* and *Oliveria decombens* essential oils was estimated by using a rat animal model for topical treatment of the DFU healing effect. The result of the experiment reveals the herbal cream possesses a remarkable ulcer healing activity with the highest tissue repair rate in comparison with other formulations. Further, the histopathological examination of the tissues indicated a higher regeneration score [41].

### 7.5 Herbal supplements

Leung and associates have performed a double-blind randomized clinical trial of an herbal drink against DFUs and its status in limb salvage. The supplement contains 12 herbs widely distributed in China, viz. *Radix astragali, Radix* *Stephaniae tetrandrae,* *Rhizoma* *Atractylodis marcocephalae, Radix rehmanniae, Radix* *Polygoni multiflori, Rhizoma dioscoreae, Radix* *Smilax china, Rhizoma alismatis, Rhizoma* *Smilacis glabrae*, *Fructus corni, Cortex moutan,* and *Fructus schisandrae.* Patients for the study were selected from orthopedic departments of two general hospitals in China based on their 7 to 12-year history of diabetes as well as a 7 to 25-week history of foot ulcers. Following the completion of the study, it was reported that the herbal supplement improved granulation tissue growth, skin temperature, and oxygen consumption around the ulcer area. Moreover, it also helps in reducing TNF-α in blood serum and plays a significant role in ulcer healing [42]. Malone and co-worker have performed a case report on the use of traditional drugs and folk medicines of Saudi Arabian for the management of DFU. A 68-year-old Bedouin female with dry gangrene was selected for the study. After the successful completion of the study, it was reported that honey and black seeds (*Nigella sativa*) followed by myrrh (*Commiphora molmol*) produced an effective result on the topical application for gangrene [43]. As part of a cross-sectional descriptive study, Bakhotmah and associates examined the complementary and alternative medicine (CAM) practices of 142 diabetics in Jeddah and Saudi Arabia. The study reported that honey (56.6%) is the most commonly used CAM for the treatment of DFU, followed by *C. molmol* (Myrrh) (37.4%), *Nigellia sativa* (Black seed) (35.1%), and *Lawsonia inermis* (Henna) (12.1%). Honey, alone or in combination with other herbal medications like black seeds and myrrh, topped the list of 10 typical natural remedies used topically to treat DFUs [44].

### 7.6 Micronutrients for the management of DFU

Micronutrients, such as trace elements and vitamins, are especially important in the treatment of DFUs because they facilitate the wound healing process. It reduces the risk of developing pressure wounds [64].

#### 7.6.1 Vitamins

Vitamin D is responsible for reducing wound fibrosis. In a study of diabetics with foot ulcers, Razzaghi, and colleagues found that providing 50,000 IU vitamin D tablets twice a week for three months aided healing by enhancing glycemic control. One of the findings was a reduction in inflammation and oxidative stress. Supplementing with vitamin D decreased parathyroid hormone synthesis, which can restrict the generation of C-reactive protein (CRP), which leads to inflammation, and reactive oxygen species, which can induce oxidative stress [64,65]. For more than half a century, vitamin E has been used to treat skin conditions and heal wounds. A study by Musalmah and co-workers investigated the efficacy of α-tocopherol as potential antioxidant agent to treat diabetic ulcers in rodents. Results from the experiment indicate that vitamin E significantly lowers plasma malondialdehyde levels and increases glutathione peroxidase activity, allowing the wounds in diabetic rats to heal faster [66]. Additionally, vitamin C is a vital component in the creation of cartilage and is a critical factor in the manufacture of collagen for the skin. Vitamin C deficiency can make wound healing more difficult and increase the risk of infection. It is also associated with antioxidant activity, collagen formation, cellular apoptosis, maturation, secretion, and breakdown during the proliferative phase. According to a recent study, vitamin C promotes the production of TGF-β1, matrix metalloproteinase-1 (MMP-1) expression, and gelatinase activity, which improves the cellular morphology of wounds [67]. In addition, vitamin A promotes wound healing at the initial stage by boosting monocytes and macrophages and moderating collagenase activity. Yet there have been no significant studies to evaluate its specific ability to heal DFUs.

#### 7.6.2 Minerals

Minerals and other trace elements play a vital role in the management of DFU. For equilibrium, the body frequently requires regular dosages in extremely tiny levels, especially during homeostasis and wound healing. In particular, minerals and trace elements serve as cofactors for wound-healing enzymes. Magnesium has been linked to a higher risk of DFU since it is a crucial cofactor for many enzymatic reactions. Further, it is also associated with impaired endothelial function, insulin dysfunction, neuropathy, and alteration of platelet function [64]. Similarly, Larijani and colleagues demonstrated that in DFU patients, serum zinc levels were significantly lower than those in other diabetic patients without foot ulcers [68]. Iron is considered one of the most important minerals which is associated with wound healing of DFU which acts as a cofactor in collagen synthesis and controls anemia. Takayama and Aoki explained the beneficial effect of lactoferrin (a glycoprotein secreted by glandular epithelial cells that bind to iron) on the initial inflammatory phase of DFU [69]. Although, few researchers have justified the importance of Magnesium, Zinc, and Iron for the quick healing of DFU other trace elements such as Boron and Copper need proper elucidation of pharmacological activity as they are involved as a cofactor in protein synthesis and collagen formation.

### 7.7 Insect products

#### 7.7.1 Propolis

Propolis is a naturally occurring anti-inflammatory resin that the worker bees consume during the process of collecting and masticating plant material. Honeybee salivary enzymes are mainly mixed with wax to produce propolis. Several studies have reported the anti-inflammatory, antioxidant, and antimicrobial properties of propolis which support its use against DFUs. Active biomolecules such as flavonoids and esters of caffeic acid commonly inhibit the matrix metalloproteinase-9 (MMP-9) and other proinflammatory proteinase which are key indicator associated with DFU [70,71]. In a clinical trial involving twenty-four patients with chronic diabetic foot ulcers for six weeks, Henshaw and his colleagues investigated the topical application of bee hive protector propolis for the treatment of DFUs. In the study, all ulcers were classified according to the established University of Texas grade (mostly grade 1) and staging system (stage B), which helps predict the ulcer healing outcomes uniformly. After the first week of treatment, the ulcer area was reduced by 41% in the propolis group, compared to 16% in the control group. Moreover, topical application of propolis significantly reduces MMP-9 of post-debridement wound fluid by 18.1% as compared to 2.8% of the control group after treatment of 3 weeks [70].

#### 7.7.2 Honey

Honey's antibacterial action is mostly due to its osmolarity, which is caused by its high sugar content, acidic pH, and presence of hydrogen peroxide. Non-peroxide components such as methylglycosal, beedefensin-1, polyphenols, and phenolic acids may explain the variance in antibacterial activity. Honey contains hydrogen peroxide in most cases, but a few do not. Consequently, some non-peroxide components may also be responsible for antibacterial activity. Manuka honey has a non-peroxide content, which is a distinct Manuka component. Kateel and co-workers in 2017, performed *in vitro* studies by considering both gram positive and gram-negative bacteria. The results of the experiment indicated that gram-positive bacteria, *Staphylococcus* *aureus*, were completely killed within 6 h of exposure to undiluted honey while gram-negative bacteria, *Escherichia* *coli*, and *Pseudomonas* *aeruginosa*, were completely killed within 8 h [72]. Similarly, in the year 2020 Chaudhary and associates evaluated the wound-healing activity of Jamun honey by using streptozotocin-induced diabetic mice model. The study revealed that topical application of Jamun honey in a diabetic mice model resulted in significant wound closure, reepithelialization, collagen deposition, and a balanced formation of myofibroblasts [73]. Furthermore, in another study conducted by Shukrimi and co-workers, a prospective clinical investigation was conduted on 30 patients (31–65 years old) to compare the efficacy of honey dressing against povidone iodine and saline dressings (control) in Wagner grade-II diabetic foot ulceration. The study revealed that the mean healing time for the honey group was 14.4 days, while 15.4 days for the standard dressing group [74]. A cross-sectional descriptive study involving 1634 DFU patients regarding the use of complementary and alternative medicine (CAM) in their daily life for the management of DFU was performed in Jeddah, Saudi Arabia. Observations have shown that more than half of the population (56.6%) uses honey as a CAM for the treatment of DFU [75,44]. Massive studies across the globe have reported the potential use of different types of honey for the treatment of DFU by performing various *in-vitro, in-vivo,* and clinical trials. The United States Food and Drug Administration (US-FDA) approved Manuka honey as a dressing for DFUs in 2007.

#### 7.7.3 Maggot debridement therapy (MDT)

Maggot debridement treatment, also known as maggot therapy, bio debridement, or larval therapy, is a 'ancient' technique of wound healing. As part of this therapy, live fly larvae are applied to wounds to aid in debridement, disinfection, and, eventually promotes wound healing. Under controlled clinical conditions, a common green bottle fly (*Phaenicia sericata*) produced myiasis in mice. This treatment is highly effective against methicillin-resistant *S. aureus* (MRSA) and other resistant bacteria. In a recent single-patient study, Azad et al. evaluated the effect of MDT on patients with DFU, one patient at a time. Eleven patients with medium wounds (size 3 cm × 11 cm) and a willingness to try natural medicinal therapy were selected. The study exposed that MDT heals the wound area (free from slough) in 30 days by promoting granulation tissues and nerve stimulations. Additionally, MDT has also succeeded in reducing wound care costs by shortening hospital stays and avoiding amputation procedures [76].

## 8 Conclusion

According to the current review's findings, the major risk of increasing foot ulceration is caused by nerve cell damage, ischemia, hypoxia, decreased angiogenesis, lack of vasodilator production, and, most likely, infection from bacteria. Furthermore, many molecular factors, including receptors, proteolytic enzymes, and neuropeptides, have been altered in this pathological condition, potentially leading to prolonged wound healing due to down-regulation of KGF, IL-10, PDGF, IGF-1, EGF, and HIF and up-regulation of TNF-α, IL-6, and TGF-β2. All of these elements and molecular markers play an important role in extending the healing of diabetic ulcerated wounds and can be used as diagnostic tools by researchers to detect patients suffering from DFU. Furthermore, the primary goal of this study is to compile all available information on *in vitro, in vivo*, case reports, and clinical symptoms utilizing natural substances to treat and manage DFU. The current review's findings provide information on the use of natural products for the management of DFU, including the utility of 18 medicinal plants with 01 isolated components, 07 polyherbal formulations such as herbal lotions, 03 insect products, and a few micronutrients such as vitamins and minerals. Despite the limited information available on the natural substance used for the management of DFU, some medicinal plants with promising pre-clinical and clinical effects reported in this article involve *Actinidia deliciosa*, *Ageratina* *pichinchensis, Aloe barbadensis, Astragalus membranaceus* var. *mongholicus, Azadirachta indica*, *Coffea* *canephora*, *Melilotus* *officinalis, Myrtus communis, Onosma microcarpum, Quercus infectoria*, *Teucrium polium,* and *Tinospora cordifolia.*

This review identified many research gaps, which may theoretically be considered legitimate information for further exploration by researchers worldwide. To begin with, although the plant extracts employed in clinical studies have significant advantages, the specific mechanism of action at the biochemical and molecular levels has less been researched, indicating research gaps. Secondly, it was discovered that relatively little research had been conducted on isolated and purified chemicals from natural sources. As a result, this remains a study gap for scientists to investigate more on the active phytoconstituents and to come up with novel molecules for the treatment of DFU. Thirdly, even though a few polyherbal formulations were documented in this study, there are relatively few marketed treatments available from alternative medicines to treat DFU. As a result, extensive clinical trials on DFU are required, taking into account the therapeutic properties of plants, animals, insects, and their active compounds. Hence, this review concludes that natural therapy can be explored further to treat and manage DFU in an efficient, cost-effective, and reproducible manner.

## Source of funding

None.

## CRediT authorship contribution

**Suman Kumar:** Methodology / Study design, writing-original draft; **Alakesh Bharali:** Investigation, Resources; **Himangshu Sarma**- Software; **Susankar Kushari:** Data curation; **Sameeran Gam**: Validation; **Iswar Hazarika**: Formal analysis; **Satyendra K. Prasad**: Writing – review and editing; **Damiki Laloo**- Conceptualization, supervision, writing-review and editing.

## Declaration of competing interest

The authors declare that they have no known competing financial interests or personal relationships that could have appeared to influence the work reported in this paper.
